# Investigating the effect of solvent vapours on crystallinity, phase, and optical, morphological and structural properties of organolead halide perovskite films†

**DOI:** 10.1039/d0ra07926j

**Published:** 2020-11-02

**Authors:** Sagar A. More, Rajendra G. Halor, Raees Shaikh, Gauri G. Bisen, Hemant S. Tarkas, Swapnil R. Tak, Bharat R. Bade, Sandesh R. Jadkar, Jaydeep V. Sali, Sanjay S. Ghosh

**Affiliations:** Optoelectronics/Organic Photovoltaics Laboratory, Department of Physics, Kavayitri Bahinabai Chaudhari North Maharashtra University Jalgaon-425001 Maharashtra India ssghosh@nmu.ac.in; Department of Physics, Savitribai Phule Pune University Pune-411007 Maharashtra India

## Abstract

A comprehensive study regarding the effect of different solvent vapours on organolead halide perovskite properties is lacking. In the present work, the impact of exposing CH_3_NH_3_PbI_3_ films to the vapours of commonly available solvents has been studied. The interaction with perovskite has been correlated to solvent properties like dielectric constant, molecular dipole moment, Gutmann donor number and boiling point. Changes in the crystallinity, phase, optical absorption, morphologies at both nanometer and micrometer scale, functional groups and structures were studied using X-ray diffraction, UV-visible absorption, FE-SEM, FTIR and Raman spectroscopies. Among the aprotic solvents DMSO and DMF vapours deteriorate the crystallinity, phase, and optical, morphological and structural properties of the perovskite films in a very short time, but due to the difference in solvent property values acetone affects the perovskite properties differently. Polar protic 2-propanol and water vapours moderately affect the perovskite properties. However 2-propanol can solvate the organic cation CH_3_NH_3_^+^ more efficiently as compared to water and a considerable difference was found in the film properties especially the morphology at the nanoscale. Nonpolar chlorobenzene vapour minutely affects the perovskite morphology but toluene was found to enhance perovskite crystallinity. Solvent properties can be effectively used to interpret the coordination ability of a solvent. The present study can be immensely useful in understanding the effects of different solvent vapours and also their use for post-deposition processing (like solvent vapour annealing) to improve their properties.

## Abstract 2

Effect of solvent vapours on the micrometer and nanometre scale morphology of CH_3_NH_3_PbI_3_.

## Graphical abstract

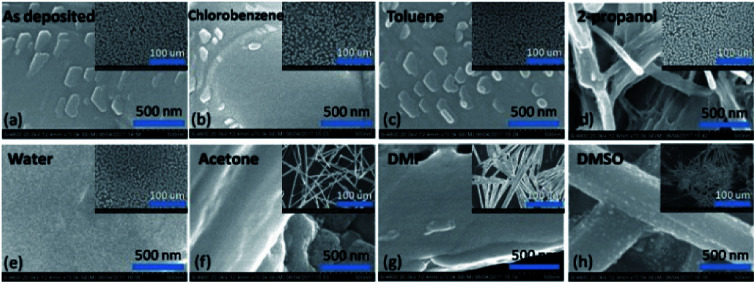

## Introduction

Organolead halide perovskites with the general formula ABX_3_ (where A is an organic cation, B a divalent metal ion, and X a halide) have potential applications in many fields such as photovoltaics,^1–3^ field-effect transistors (FETs),^4,5^ light-emitting devices (LEDs),^6,7^ lasers,^8^ and photodetectors.^9,10^ This class of materials has several advantages like solution processability, low-temperature processing requirements, flexibility in terms of composition, and it can be deposited on flexible substrates.^11–13^ Organolead halide perovskites combine the properties of inorganic materials (such as high carrier mobility and a wide band gap range from ∼1.1 to ∼4 eV) and those of organic materials (such as structural diversity, high-efficiency luminescence, and plastic mechanical properties).^14–16^ Improved device performances can be achieved by obtaining enhanced optoelectronic properties of the perovskite materials through various methods and by understanding their properties in a better way. The only aspect of organic–inorganic perovskites which hinders the journey to the commercial market is their instability.^17^ Both intrinsic and extrinsic factors may be responsible for the instability. Prominent among the intrinsic factors are thermal, photochemical, and degradation due to ion migration. Various groups have studied the effect of using different solvents dimethyl sulfoxide (DMSO), dimethylformamide (DMF), γ-butyrolactone, dimethylacetamide, (*N*-methyl-2-pyrrolidone), and their mixtures for perovskite film formation.^18,19^ It is reported that the perovskite films are formed through complex intermediates from the precursor solution of DMF, DMSO, and other solvents. Radicchi *et al.* have investigated the chemistry of typical precursor solutions employed for lead halide perovskite synthesis by a combined experimental and computational approach.^20^ Many groups have studied the effect of humidity and oxygen on the perovskites.^20–22^ Hao Xiong *et al.* have studied solvent vapour annealing of oriented PbI_2_ films for improved crystallization of perovskite films in the air.^23^ Jun Luo *et al.* studied the mechanism and effect of γ-butyrolactone solvent vapour post-annealing on the perovskite of a mesoporous solar cell structure.^24^ However, a complete study regarding the impact of solvent vapours of different classes on the organolead halide perovskite properties is not present in literature.

Different types of solvents are commonly available in the laboratory, and while synthesizing and fabricating the devices, solvent vapours can be available in the surroundings. Therefore, it is important to know the effect of these commonly available solvents of different classes on the organolead halide properties. Also understanding the use of solvent vapours for post-deposition processing (like solvent vapour annealing) to improve perovskite properties. To the best of our knowledge, there is no comprehensive report where the effect of vapours of commonly available solvents from different classes *viz.* polar protic, polar aprotic and non-polar which can have varied effects and also to different levels on perovskite properties has been studied. In the present work, we have studied the impact of exposing vapours of various solvents like dimethylformamide (DMF), dimethylsulfoxide (DMSO), acetone, water, 2-propanol, chlorobenzene and toluene on the perovskite material for relatively short time up to 30 minutes. We have studied the effect of solvents on absorption, crystallinity, phase, morphology both at the nanometre and micrometre scale and the structure of perovskite. Our results show that vapours of polar protic solvents like DMF and DMSO affect the optical properties, morphology, and crystalline structure of perovskite drastically in a short time. Acetone and the polar protic water and 2-propanol affect some aspects of the perovskites. Vapours of non-polar solvents like chlorobenzene and toluene have a minimal effect on the perovskite properties. Toluene vapour was found to improve the perovskite crystallinity without much affecting the nanoscale morphology. We also propose the use of mixed solvents to obtain tailor-made properties of solvents for obtaining desired properties by vapour exposure.

## Materials and methods

All the chemicals and solvents were purchased from Sigma Aldrich. CH_3_NH_3_I (MAI) was synthesized in the laboratory, and the synthetic details are provided in the ESI.†

### CH_3_NH_3_PbI_3_ film deposition

CH_3_NH_3_PbI_3_ film was deposited using a single step spin coating method. A mixture of precursors MAI (3 M) and PbCl_2_ (1 M) was prepared in DMF. The solutions were stirred at 40 °C for 2 hours, and after that, spin coated on a glass substrate at 3000 rpm for 1 minute. The spun films were then heated at 100 °C for 45 minutes.

### Pristine PbI_2_ film deposition

PbI_2_ film was deposited using spin coating. A PbI_2_ solution (461 mg mL^−1^) was prepared in DMF, and this solution was stirred at 70 °C for 2 h. Then, this solution was spin-coated on pre-cleaned glass substrates at 3000 rpm for 1 minute.

### Solvent vapour exposure

**Fig. 1:**
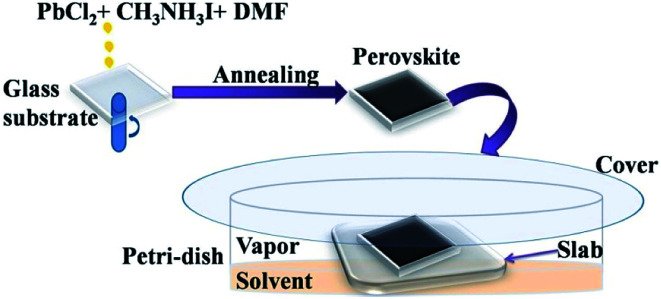
Schematic diagram showing the perovskite film formation and exposing to the solvent vapours.

The glass substrates coated with perovskite films were placed in Petri-dish on thicker platforms made by staking of 5 glass slides. 10 mL of required solvents were then poured in each Petri-dish (90 mm diameter). Precautions were taken so that the solvent does not touch the film directly. The Petri-dish was then covered with another glass plate. A schematic diagram showing the film formation and vapour exposure procedure is given in the Fig. 1.

### Characterizations

UV-visible absorption spectrophotometer (UV 1600, Shimadzu) was used to measure the absorption spectra of the samples. Field Emission Scanning Electron Microscope (FE-SEM Hitachi-4800) was used to study the surface morphological changes of the samples by applying an accelerating voltage of 20 kV. D8 advance Bruker X-ray diffractometer with incident source wavelength *λ* = 1.54 Å in coupling mode was used for the identification of material phase and study the effect on crystalline properties. Fourier transformed infrared (FTIR) spectrometer (PerkinElmer) was used to examine the molecular nature of the sample constituents. Raman spectra were recorded by using (Renishaw inVia microscope Raman, resolution 1 cm^−1^, excitation source 632.8 nm line of He–Ne laser). Weight of the amount of solvent settled on the exposed film was measured using Shimadzu balance AUW220D with readability 0.01 mg.

## Results

The following equations govern the reaction between PbCl_2_ and CH_3_NH_3_I in the solution and thereafter during the perovskite phase formation due to thermal annealing after spin coating.
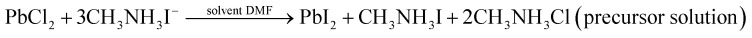

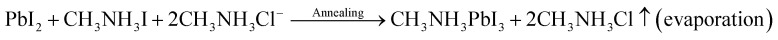


In the precursor complex, PbI_2_ is formed due to an ion exchange between PbCl_2_ and CH_3_NH_3_I. After thermal annealing, it is converted into the CH_3_NH_3_PbI_3_ phase. There are numerous reports which show that CH_3_NH_3_Cl evaporates during thermal annealing process.^25^ Several studies have suggested that the presence of CH_3_NH_3_Cl slow down the film formation process during thermal annealing which therefore results in more crystalline and stable perovskite.^26^ Due to this reason PbCl_2_ was preferred in place of PbI_2_ for perovskite film formation in the present study.

### X-ray diffraction study

**Fig. 2:**
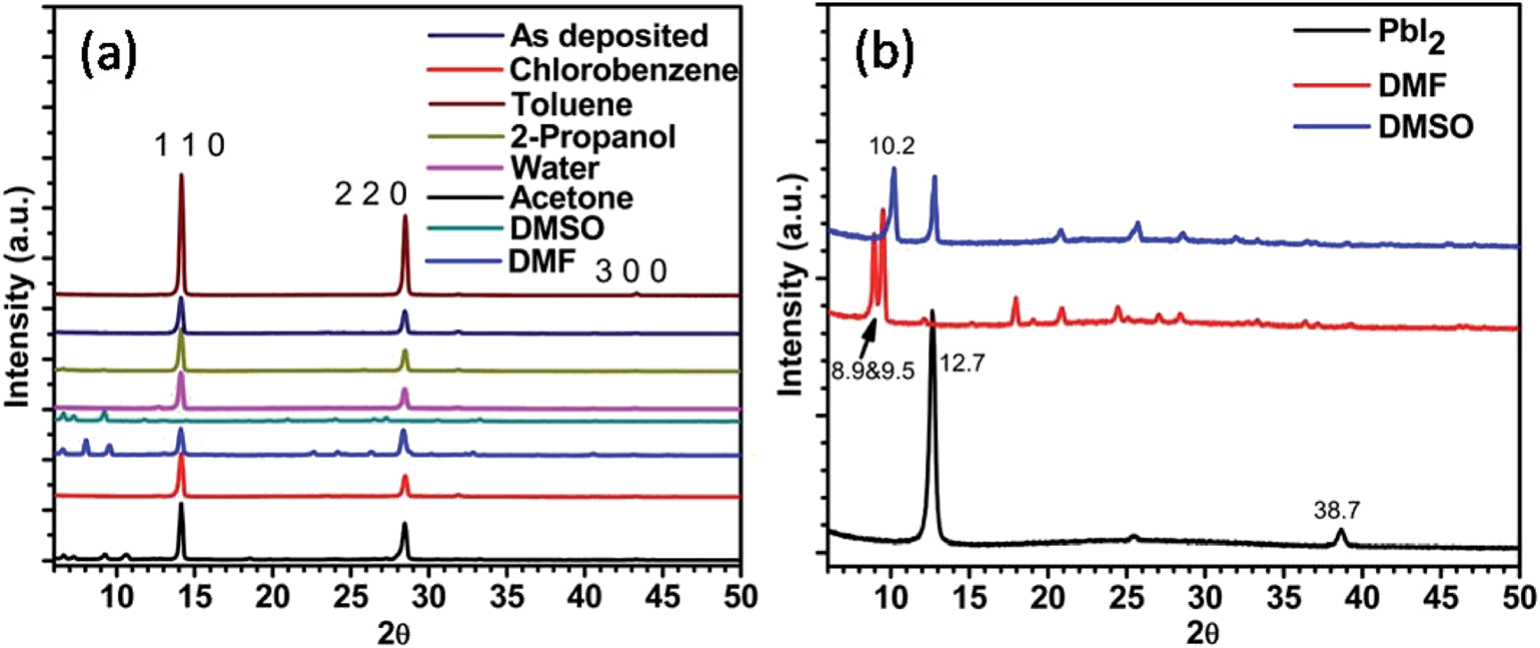
X-ray diffraction pattern of (a) perovskite film before and after exposure to different solvent vapours, (b) PbI_2_ films before and after exposure to different solvent vapours.

X-ray diffraction (XRD) pattern of the annealed perovskite film and of those after exposing to different solvent vapours for 30 minutes were recorded (Fig. 2). It shows that, good phase of CH_3_NH_3_PbI_3_ is formed after thermal annealing the spin coated film. Standard diffraction peaks at 14.2°, 28.5° and 43.3° values of 2*θ* are attributed to (1 1 0), (2 2 0), and (3 0 0) planes respectively.^27,28^ No signatures of other phases containing chlorine were detected. On exposing the films to different solvent vapour many changes are introduced in the XRD pattern. Few details are given in Table 1. On exposing to DMSO vapour the standard peaks corresponding to perovskite were completely missing. However, many low intensity peaks were observed, prominent among them were at 6.6°, 7.3° and 9.3° values of 2*θ*. These peaks were attributed to intercalation of DMSO or a mixture of DMSO and the organic part into PbI_2_ phase. To confirm whether this is due to the intercalation of only DMSO or DMSO plus organic part into the PbI_2_ phase, we exposed pristine PbI_2_ film with DMSO vapour. In addition to standard PbI_2_ peak at 12.7° a new peak at 10.16° value of 2*θ* was observed (Fig. 2b). Therefore it is expected that the other peaks at 6.6°, 7.3° and 9.3° values of 2*θ* in the DMSO vapour exposed perovskite film are due to the intercalation of DMSO and organic component of the perovskite into the PbI_2_ lattice. This is in accordance with previously reported literature.^29^ This shows that PbI_2_ and organic component are formed due to the exposure of DMSO vapour. The quality of this PbI_2_ is however different from that of pure PbI_2_ film. This is in accordance with the absorption studies shown in the next sub-section. The perovskite film exposed to DMF vapour shows less intense standard perovskite peaks. Additionally peaks at 6.6°, 8.13° and 9.5° values of 2*θ* were observed. The PbI_2_ film shows additional peaks at 8.9° and 9.5° after exposing with DMF vapour. Therefore the peaks at 6.6° and 8.13° are attributed to the intercalation of DMF and organic part into PbI_2_. For acetone exposed perovskite film, additional peaks were observed at 6.6°, 7.3°, 9.3° and 10.6°. Acetone vapour exposed PbI_2_ film does not show any peak at lower 2*θ* values. Thus all the peaks at lower 2*θ* values in the perovskite film are due to the intercalation of acetone and organic part into PbI_2_. Above results indicate that the polar aprotic solvent vapour exposure leads to the formation of intercalation state or complex between solvent, PbI_2_, and the organic part.

**Table 1:** Additional XRD peaks observed in the perovskite films upon exposing to different solvent vapours are shown

Solvent vapour	2*θ* (degree) values of additional peaks observed upon solvent vapour exposure
DMSO	6.6, 7.3, 9.3 and 11.9
DMF	6.6, 8.1 and 9.6
Acetone	6.6, 7.3, 9.3 and 10.6
Water	12.7
2-Propanol	6.6, 7.3, 9.3
Chlorobenzene	No additional peaks
Toluene	No additional peaks

Water vapour exposure for 30 minutes on perovskite film shows a new peak at 12.7°. This peak is attributed to PbI_2_ (0 0 1) phase.^25^ This shows that upon exposing to water vapour the beginning of PbI_2_ formation takes place in 30 minutes time. The standard perovskite peaks were also seen which indicates that complete conversion of perovskite into PbI_2_ does not take place in this time scale. The perovskite film exposed to 2-propanol vapour showed very low intensity peaks in the lower 2*θ* values. In analogy with studies on DMF and DMSO vapours these are also attributed to the intercalation of solvent and organic component into the PbI_2_ phase. Upon exposure with chlorobenzene, no prominent changes in the XRD pattern were observed. However by exposing with non-polar toluene solvent the intensity of main peak improves. This shows enhancement in grain size of perovskite upon exposing with toluene vapour. The crystallite size ‘*d*’ was calculated using the following Scherer formula:*d* = 0.9*λ*/*β* cos *θ*,where *β* is the full width at half maximum.

For the as prepared film crystallite size calculated was 28.8 nm while after exposing to toluene it improved to 32.1 nm.

### UV-visible absorption spectroscopy

**Fig. 3:**
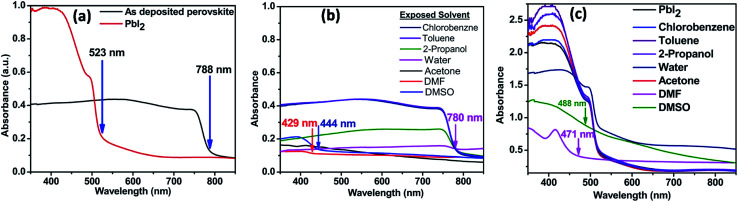
UV-visible absorption spectra of (a) annealed perovskite film and PbI_2_ film (b) perovskite films after exposure to different solvent vapours (c) PbI_2_ films after exposure to different solvent vapours.

Fig. 3a shows the UV-visible absorption spectra of the annealed perovskite and PbI_2_ film. Optical bandgap was calculated by using the following relation:
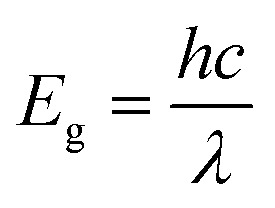
Where *h* is the planks constant, *c* is the speed of light in vaccum and *λ* is the absorption edge.

Tabulated values of absorption edge and corresponding calculated optical bandgap are shown in Table 2. It shows the absorption edge at around ∼788 nm for CH_3_NH_3_PbI_3_, while that for PbI_2_ is at ∼523 nm corresponding to optical bandgap of 1.57 eV and 2.37 eV respectively. The absorption spectrum matches with those given in the literature.^30^Fig. 3b shows the absorption spectra of CH_3_NH_3_PbI_3_ films after exposing to different solvent vapours for 30 minutes. The absorption edge remains same for films exposed to 2-propanol, toluene, and chlorobenzene vapours. It decreases slightly to 780 nm when exposed to water vapour. This indicates an increase in optical bandgap due to the partial conversion to PbI_2_, which is indicated in XRD result also. Minimum changes in films exposed to toluene and chlorobenzene vapours were observed. This shows that there are nearly no changes in the absorption properties of the films upon exposing to these vapours. For the films exposed to DMF, DMSO, and acetone vapours, the absorption spectra change remarkably. DMF vapour exposed film shows blue shifted absorption spectra compared to that of exposed PbI_2_ film exposed to the same vapour. Fig. 3c shows the absorption spectra of PbI_2_ film exposed to different vapours. This signifies that by exposing to DMF vapour, perovskite breaks down partially into PbI_2_ and the organic components giving rise to blue shift. This is again confirmed by the change in colour of perovskite film from black to faint yellow and thereafter transparent after exposure to DMF vapour. The colour of PbI_2_ film exposed to DMF vapour also turns transparent from initially yellow colour. Film exposed to DMSO vapour also shows blue shifted absorption spectrum compared to that of exposed PbI_2_ film. Also the colour of film becomes faint yellow initially and then transparent. It can be therefore said that by exposing to DMSO vapour also perovskite breaks down to PbI_2_ and organic components. The perovskite film which is exposed to acetone vapour shows absorption property completely different from the pure perovskite film. A closer look at the spectrum reveals that it is similar to pristine PbI_2_ film with small peaks having reduced absorption at 515 nm, 455 nm and 415 nm. This shows that by exposing the perovskite film to acetone vapour also PbI_2_ and organic components are formed. The decrease in absorption is assigned to the partial dissolution of formed PbI_2_ in acetone. No changes were observed in the pristine PbI_2_ absorption spectrum after exposing to acetone. This shows that PbI_2_ becomes soluble in acetone due to the presence of organic component in perovskite. This is due to strong interaction of I^−^ with PbI_2_.

**Table 2:** Absorption onset and calculated bandgap has been tabulated

Solvent vapour	Absorption edge (nm)/calculated bandgap (eV)	
	Perovskite	PbI_2_
Unexposed	788/1.57	523/2.37
DMSO	444//2.79	Broad edge 488/2.54
DMF	429/2.89	471/2.63
Acetone	Clear edge not seen	523/2.37
Water	780/1.59	523/2.37
2-Propanol	788/1.57	523/2.37
Chlorobenzene	788/1.57	523/2.37
Toluene	788/1.57	523/2.37

The absorption coefficient reduces (2.4 × 10^4^ cm^−1^ for perovskite to 1.4 × 10^4^ cm^−1^ for 2-propanol and 0.2 × 10^4^ cm^−1^ for water vapour exposed samples around the absorption edge) for perovskite film exposed to water vapour and to a lesser extent in film exposed to 2-propanol vapour. This reduction in absorption coefficient by exposing to water vapour may be due to the conversion of perovskite to hydrated forms. It has been reported previously that perovskite can form (CH_3_NH_3_)_4_PbI_6_·2H_2_O due to water vapour exposure.^31^ The changes in absorption spectra of the film exposed to 2-propanol vapour also shows that the perovskite properties changes.

### Raman spectroscopy

**Fig. 4:**
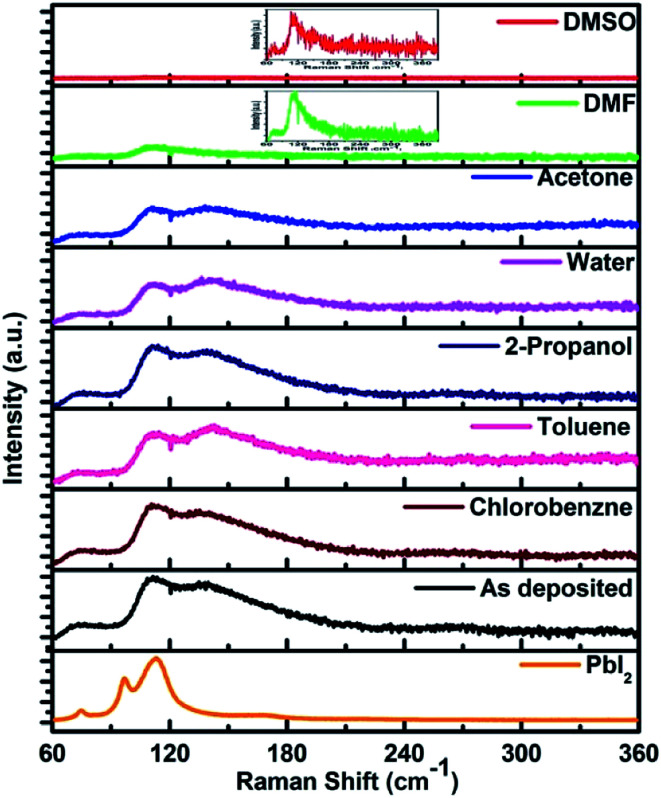
Raman spectra of the PbI_2_ and perovskite films before and after exposing to different solvent vapours.

The above discussion was further supported by Raman spectroscopic studies. The excitation wavelength used in the present study is 632.8 nm which is near resonance for Raman measurement of lead halide perovskite. We found assortment in literature in assigning the Raman bands for CH_3_NH_3_PbI_3_ material. The Raman spectrum for pure PbI_2_ (2*H*-polytype) is shown in Fig. 4. The most dominating peak corresponding to PbI_2_ is at 113 cm^−1^. For the films unexposed to any vapour, Raman bands obtained at 71, 110 cm^−1^ were attributed to the Pb–I cage in CH_3_NH_3_PbI_3_ (Fig. 4). The band at 138 cm^−1^ is attributed to the libration motion of methyl ammonium (MA) cation. The bands at 260 and 350 cm^−1^ can be assigned to the MA cation torsional mode of CH_3_NH_3_PbI_3_. Raman bands obtained in the present study, their assignment and comparison with literature is given in Table 3.

**Table 3:** Different peaks in the Raman spectra found in the present study and that given in literature are tabulated and assigned

Sr. no.	Observed peak position for CH_3_NH_3_PbI_3_ in cm^−1^	Peak positions given in literature^31–34^ for CH_3_NH_3_PbI_3_ in cm^−1^	Assignment
1	71	69, 71	Pb–I stretching
2	110	110	Pb–I stretching
3	138	138	Libration of organic cation
4	260	250	MA torsion mode
5	350	348	MA torsion mode

The Raman spectrum for each perovskite thin film after exposing to different vapours for 30 minutes are also shown in the Fig. 4. Film exposed to DMF vapour shows only two bands at 71 and 110 cm^−1^. In addition the band at 110 cm^−1^ was with much reduced intensity than that in the unexposed film. The bands at higher wavenumbers were completely absent after exposing the film to DMF vapour. If the light source intensity and the wavelength of the source are kept same the Raman peak intensity is mainly affected by concentration of the sample or number of molecules and the scattering properties of the sample.^35,36^ Absence of the peaks at 138, 260 and 350 cm^−1^ which are corresponding to the modes of organic part of the perovskite, indicates that the concentration of number of scattering molecules and their scattering property is reduced. This indicates towards the dissolution of organic part of CH_3_NH_3_PbI_3_ due to the solubility in DMF. The presence of the peak at 110 cm^−1^ indicates to the conversion to the PbI_2_ phase. The reduced intensity of this peak points to the dissolution of the PbI_2_ phase also in the solvent. A small shift of 3 cm^−1^ of this peak towards lower wave-number compared to 113 cm^−1^ of the pure PbI_2_ phase indicates the increase in bond length and hence the weakening of the bond. The quality of PbI_2_ is not same to the pure PbI_2_ film due to the intercalation of organic matter and solvent. This is in agreement with the XRD results shown. These results show the ability of DMF to dissolve both the organic and inorganic counterparts of CH_3_NH_3_PbI_3_. Similar results were obtained by exposing the films to DMSO vapour. The band at 110 cm^−1^ was broader than the DMF vapour exposed film implying a highly disordered structure of PbI_2_ formed.

In case of acetone vapour exposure, all the bands for the perovskite were observed, but with slightly different intensities. Small broadening of the peaks corresponding to Pb–I cage at 110 cm^−1^, and that of peak corresponding to MA libration mode at 138 cm^−1^ suggests the partial and selective weakening of the bonds corresponding to these modes. In case of 2-propanol vapour exposure, we observed very small changes in the band intensity. Exposing the film to chlorobenzene vapour, no changes in the Raman spectra were observed. Toluene vapour exposed film shows peak shift from 138 cm^−1^ to 142 cm^−1^. This shift towards higher wavenumber indicates shortening of the bond length. Bond length is inversely proportional to the bond strength.^37^ This shift therefore indicates strengthening of the bond corresponding to the libration mode of the MA cation. A lot of research has been done on the degradation of perovskite by water/humidity.^38,39^ We found that after 30 minutes of exposure perovskite shows a very small decrease in intensity corresponding to Pb–I cage and broadening of CH_3_NH_3_PbI_3_ cage bands.

### Scanning electron microscopy

**Fig. 5:**
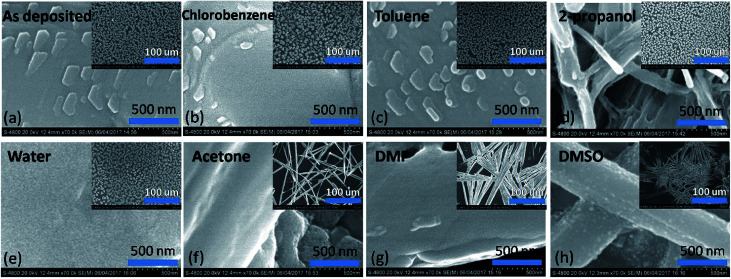
Scanning electron microscope images in both the nanoscale and microscopic scale of the unexposed and solvent vapour exposed perovskite films.

Fig. 5 shows the scanning electron microscope images of perovskite film and that by exposing to different solvent vapours for 30 minutes each. Drastic changes were found in morphology after exposure to DMSO and DMF. Broom like structures are seen in the films in the micrometre scale. Widths of the structures being much smaller in the DMSO vapour exposure film. Similar trend was observed at the nanometre scale images. Acetone vapour exposed film also shows drastic changes in the film morphology. The effect of water vapour shows changes in the shape of the islands found in the film. At the nanometre scale features disappeared in the film which were visible in the as deposited film. 2-Propanol vapour shows changes in the size of islands and voids were found in each individual island increasing the porosity of the film. Minimum effect was found by exposing the films to chlorobenzene and toluene both at the nanoscale as well as micrometre scale. However, as per the results of previous sections it is found that toluene exposure improves the crystallite size. This may be due to very small solubility which is just sufficient to minutely dissolve the small boundaries and thereby fusing them.

### Fourier transform infrared spectroscopy

**Fig. 6:**
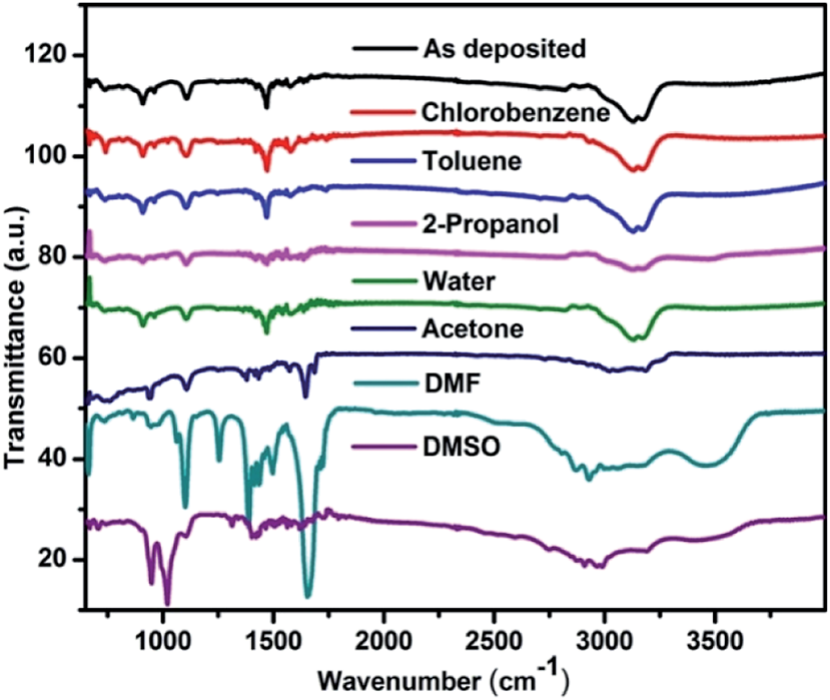
FTIR spectra of the perovskite films before and after exposing to different solvent vapours.

FTIR spectra in the range between 4000 cm^−1^ to 400 cm^−1^ were recorded which belongs to the absorption of organic materials.^40^ Therefore the effect of solvent vapour on the organic part of the perovskite can be explained by using FTIR spectroscopy. Fig. 6 shows the FTIR spectra of CH_3_NH_3_PbI_3_ before and after exposure to various solvent vapours. The main vibrational lines in the spectra can be assigned to fundamental modes of the CH_3_NH_3_ cation and anharmonic combinations thereof. Three characteristics are commonly examined in the FTIR spectra: peak position, integrated peak intensity, and peak width. The peak positions provide a fingerprint that can be used to identify chemical groups. The integrated intensity is proportional to the concentration of absorbing bonds.^40^ The peak width is a function of the homogeneity of the chemical bonding. Table 4 shows the characteristic vibrational bands of CH_3_NH_3_PbI_3_ obtained in the present study and that of reported in the literature. All NH_3_ related peaks are stronger than the CH_3_ vibrations, mainly due to the positive charge located on the ammonium group. After exposing the films to toluene and chlorobenzene vapour no changes in the vibrational band structures like shift in peak position, integrated peak intensity and peak width were observed. This shows that the vibrational modes, their concentration and the homogeneity remain intact by exposing with these vapours. Since the peak width remains same it implies that defects and bond strains are not introduced in the functional groups. Upon exposing with water vapour no change in the FTIR spectrum was observed. However there are reports where changes in the FTIR spectrum have been reported.^42^ These are however for long time exposure to water vapour. It can therefore be concluded that water vapour exposure for this time scale is not sufficient to bring major changes in the methyl ammonium part of the material.

**Table 4:** Various vibrational bands observed in the present FTIR study and in reported literature and their assignment has been tabulated

Vibrational bands observed	Vibrational bands reported^40,41^	Assignment for CH_3_NH_3_PbI_3_
908	911	CH_3_NH_3_^+^ rocking
960	960	C–N stretching
1421	1425	AsymCH_3_ bend
1469	1469	SymNH_3_^+^ bend
1577	1577	AsymNH_3_^+^ bend
3000	3005	C–H stretch
3100	3105	N–H stretch (sym)
3130	3132	SymNH_3_^+^ stretch
3170	3179	AsymNH_3_^+^ stretch

Upon exposing with 2-propanol and acetone vapour the symmetric and asymmetric NH_3_ stretch integrated peak intensity decreases. This indicates that the concentration of these absorbing bonds reduces upon these solvent vapour exposures. The intensity corresponding to CH_3_–NH_3_^+^ rocking, symmetric and asymmetric NH_3_^+^ bend and C–N stretching vibrational modes also reduces which indicates that the concentration of these groups also reduces upon solvent vapour exposure. This is an indication of the dissolution of organic counterpart of the material. Upon acetone vapour exposure a peak at 1645 cm^−1^ appears, the intensity of which increases with exposure time. Acetone having the carbonyl (C=O) functional group shows an intense peak at 1715 cm^−1^ wavenumber.^40^ The shift in this peak is therefore due to change in chemical environment corresponding to this functional group due to the interaction with perovskite.

When the perovskite samples were exposed to DMF vapour, we see changes in the vibrational band within very short period of time (ESI†). The symmetric and asymmetric CH_3_ and NH_3_ stretch peaks becomes broad. This suggests that the homogeneity of these chemical bonds is disturbed due to defects and bond strain. Small shift in bond strengths cause small shifts in peak positions. The net result is broadening of the absorption band. After 30 minutes exposure the peaks disappears. In the finger print region many new peaks appears in the spectrum corresponding to DMF. Similar results were observed for the DMSO vapour exposed film also.

## Discussion

CH_3_NH_3_PbI_3_ is an ionic crystal in which the organic part CH_3_NH_3_^+^ is the cation with +1 oxidation state, the metal ion Pb^2+^ has +2 oxidation state while the halogen I is the anion with −1 oxidation state.^43^ Crystals of this perovskite are held together by ionic interactions between the organic and inorganic portions as well as hydrogen-bonding interactions between the hydrogens in NH_3_^+^ head and the iodine atoms. The hydrogen bonding interactions between the organic cation and halide ions of the perovskite lattice provide structural stability. The effect of particular solvent vapour on the perovskite properties can be explained by considering the solvent properties like dielectric constant, molecular dipole moment, Gutmann donor number, boiling point *etc.* Few properties of the solvents used in the present study have been given in Table 5 below. Aprotic polar solvents lack O–H or N–H bonds and so do not have hydrogen bonding.^44^ However, due to the presence of carbonyl or sulfoxide groups they can act as hydrogen bond acceptors. The Pb^2+^ and CH_3_NH_3_^+^ cations in CH_3_NH_3_PbI_3_ will have the ability to readily bond with the partially negative oxygen, nitrogen or sulphur atoms in aprotic solvents. This bonding enables dissociation of the PbI_6_ octahedral frame that forms the skeleton of the organic–inorganic perovskite material, thereby resulting in high solubility of the perovskite material in polar aprotic solvents. Therefore, the aprotic solvents have the ability to form PbI_2_-solvent compounds.

**Table 5:** Solvent properties are tabulated^45,46^

Solvent	Dielectric constant	Dipole moment (*D*)	Gutmann donor number^44^ (kcal mol^−1^)	Boiling point (°C)
DMSO	47.2	3.96	29.8	189
DMF	36.7	3.82	26.6	153
Acetone	20.7	2.88	17	56
2-Propanol	10.9	1.58	21.1	82.5
Water	80.1	1.85	18	100
Chlorobenzene	5.62	1.69	3.3	132
Toluene	2.38	0.37	0.1	110.6

Inspite of the fact that DMSO, DMF and acetone are polar aprotic solvents there is a difference in the effects their solvent vapours have on the perovskite film properties. In general, higher solvent polarity enables better solubility of ionic compounds. DMSO has the largest value of dipole moment and dielectric constant followed by DMF and then acetone. It is therefore perovskite films exposed to DMSO vapour shows no XRD peaks corresponding to CH_3_NH_3_PbI_3_. The combined study of UV-visible absorption, XRD and Raman spectroscopy suggested the formation of solvent and organic component intercalated PbI_2_ in the DMSO vapour exposed films. Also the bands corresponding to organic component were completely missing in FTIR and Raman spectra. The effect of acetone vapour was minimal due to its low polarity among the polar aprotic solvents. In addition DMSO has the higher value of Gutmann donor number compared to DMF and therefore coordinating ability of DMSO is greater. The Gutmann donor number is a quantitative measure of Lewis basicity and gives the ability of a solvent to solvate cations and Lewis acids.^45,47^ It is defined as the negative enthalpy value for the 1 : 1 adduct formation between a Lewis base and the standard Lewis acid antimony pentachloride, in dilute solution in the non-coordinating solvent 1,2-dichloroethane with a zero donor number. The donor number of acetone is much smaller than that of DMSO and DMF. This is an indication of the reduced ability of acetone to solvate the Pb^2+^ and CH_3_NH_3_^+^ cation as compared to the other two polar aprotic solvents. Our experiments have shown that acetone is not a good solvent for PbI_2_. However, when CH_3_NH_3_I is added, PbI_2_ becomes readily soluble in acetone. It suggests that as CH_3_NH_3_PbI_3_ comes in contact of acetone vapours, it dissolves the organic part and results in intercalation of acetone and organic component in PbI_2_. Several new XRD peaks in the perovskite film after exposing to acetone vapour confirms this. FTIR study confirms the fact that acetone easily affects the organic part in CH_3_NH_3_PbI_3_. The above discussion indicates that DMSO has the strongest coordination capability with the Pb^2+^ and CH_3_NH_3_^+^ cations in CH_3_NH_3_PbI_3_. This coordination ability can be as a monodentate ligand by donating a pair of electrons to the cations through atom of the S–O bond and also by hydrogen bonding.^48^ Therefore DMF seems to be a weaker field ligand compared to DMSO but stronger than acetone. For the aprotic solvents therefore, the solvent coordination ability follows the same trend as its Gutmann donor number.

In addition to the solubility factor, acetone have comparatively low boiling point compared to DMF and DMSO. It is therefore, once deposited, DMF and DMSO does not evaporate easily from the perovskite surface. This results in more effective dissolution of perovskite and therefore reduction in standard perovskite peak intensity. This effect is most prominent in the case of DMSO which has the highest boiling point of 189 °C as compared to 153 °C for DMF. To confirm this, the exact amount of solvent settled on the exposed film was measured by weighing the coated film before and after exposing with the specific solvent vapours. Results are given in Table 6. It clearly shows that solvents with high boiling point stick more readily to the film after settling on the surface. In the process of dissolution, solute separates into ions or molecules, and molecules of solvent surround each ion or molecule. More the number of molecules, more the dissolution expected.

**Table 6:** Amount of solvent deposited on the exposed film after 30 minutes is given

Solvent/molecular weight	Weight of solvent after exposing to solvent vapours (mg)/number of moles of solvent
DMSO/78.13	0.90/1.15 × 10^−5^
DMF/73.09	0.66/9.03 × 10^−6^
Acetone/58.08	0.07/1.20 × 10^−6^
Water/18.01	0.63/3.50 × 10^−5^
2-Propanol/60.10	0.15/2.47 × 10^−6^
Chlorobenzene/112.56	0.85/7.55 × 10^−6^
Toluene/92.14	0.17/1.84 × 10^−6^

2-Propanol and water used in the present study are polar protic solvents that possess O–H bonds, and so they can participate in hydrogen bonding. These solvents can also serve as acids (sources of protons) and weak nucleophiles (forming bonds with strong electrophiles). Due to the H bonding present in these solvents, anion I^−^ can be solvated. The solubility of PbI_2_ is very low in these solvents. This is confirmed by the fact that no changes were observed in the absorption and XRD pattern of PbI_2_ film exposed to these solvent vapours. However, considerable changes were observed in the CH_3_NH_3_PbI_3_ films due to these solvent vapours. Even though both these solvents are polar protic, the degradation mechanism upon exposure to their solvent vapour seems to be different. Negligible or no changes were observed in the FTIR spectra of film exposed to water vapour for 30 min. This shows no changes take place in the methylammonium ion. Raman spectroscopy results show a slight decrease in peak intensity corresponding to Pb–I stretching and libration mode of organic cation, which indicates the decrease in the number of moieties corresponding to these modes. It also shows a decrease in CH_3_NH_3_^+^–I^−^ hydrogen bond and Pb_2_^+^–I^−^ bonds. Water is a polar solvent with high dielectric constant and strong hydrogen bonding. Water vapour can, therefore, weaken the above hydrogen bonds in CH_3_NH_3_PbI_3_, leading to the formation of a small amount of PbI_2_ (As shown in the XRD result) by liberating some CH_3_NH_3_^+^ and I^−^. X-ray diffraction pattern of 2-propanol vapour exposed perovskite films show new very low-intensity peaks in lower 2*θ* values. We attribute this to the intercalation of the organic component of perovskite into the PbI_2_ lattice. This effect is similar but small in magnitude as compared to that of aprotic solvents. This effect was not observed for perovskite film exposed to water vapours. This is due to the ability of 2-propanol to solvate the organic component (as shown in the FTIR spectra). 2-Propanol has a lower polarity compared to water but a higher Gutmann donor number. This is an indication of higher coordination ability of organic cation by 2-propanol as compared to water, inspite of more number of water vapour molecules settled on the film as shown in Table 6.

Both chlorobenzene and toluene have lower values of dipole moment and very low values of Gutmann donor numbers; toluene has lower numbers than chlorobenzene. This shows a very weak ability of toluene to affect the ionic perovskite molecule. Crystallinity changes indicate that toluene has at least some ability to dissolve CH_3_NH_3_PbI_3_. This weak ability is helpful by weakly dissolving the perovskite and thereby healing the interface between grain boundaries at the nanoscale and enhancing the crystallinity. The increased XRD peak intensity indicates this in the film exposed to toluene vapour. It is observed that the coordination ability of different solvents with the cations of CH_3_NH_3_PbI_3_ follows the same trend as the Gutmann donor number. Thus donor number is an important parameter and can be used to interpret the coordination ability of a solvent, with perovskite materials.

## Conclusions

We have therefore produced a comprehensive study of the effect of various solvent vapours of different classes on the perovskite properties. CH_3_NH_3_PbI_3_ is ionic and can interact with different solvents, the magnitude of which depends on the solvent properties. We showed that aprotic solvents like DMSO and DMF considerably deteriorates the perovskite properties in a very short time due to their high molecular polarity, Gutmann donor number and high boiling point. Acetone also being an aprotic solvent deteriorates the perovskite properties but moderately, due to its limited ability to solvate the cation and interaction with the polar solute. Water and 2-propanol are both polar protic solvents but interacts differently with perovskite. Due to the higher Gutmann donor number of 2-propanol, it can solvate the organic cation CH_3_NH_3_^+^ more efficiently as compared to water. Having very low values of molecular dipole moment and Gutmann donor numbers, chlorobenzene and toluene are not able to affect perovskite much. The extremely weak ability of toluene vapour to interact with perovskite was helpful in fusing the neighbouring grains and heals the perovskite defects at the nanometre scale to improve the crystallinity. The effect of different solvent has been correlated to the solvent properties like dielectric constant, molecular dipole moment, Gutmann donor number, and boiling point. We show that donor number is an important parameter and can be used to interpret the coordination ability of a solvent with perovskite materials. The present study can be useful in understanding the negative effect of different solvent vapours and also the use of solvent vapours for post-deposition processing (like solvent vapour annealing) to improve their properties. We propose the use of mixed solvents to obtain tailor-made properties of solvents to modify the perovskite properties by vapour exposure.

## Conflicts of interest

There are no conflicts to declare.

## Supplementary Material

RA-010-D0RA07926J-s001
