# Supplementary material for: Investigating the effect of solvent vapours on crystallinity, phase, and optical, morphological and structural properties of organolead halide perovskite films
Source: RSC Adv. 2020 Nov 2;10(66):39995–40004. doi: 10.1039/d0ra07926j (PMC9057470; doi:10.1039/d0ra07926j)

Electronic Supplementary Material (ESI) for RSC Advances.
This journal is © The Royal Society of Chemistry 2020

Supplementary information

Investigating the effect of solvent vapours on crystallinity, phase, optical, morphological and structural properties of organolead halide perovskite films

Sagar A. More[a], Rajendra G. Halor[a], Shaikh Raees[a], Gauri G. Bisen[a], Hemant S. Tarkas[a], Swapnil R. Tak[a], Bharat R. Bade[b], Sandesh R. Jadkar[b], Jaydeep V. Sali[a], Sanjay S. Ghosh[a]*

[a]Optoelectronics laboratory, Department of Physics, Kaviyitri Bahinabai Chaudhari North Maharashtra University, Jalgaon-425001, Maharashtra, India.
[b]Department of Physics, University of Pune, Pune-411007, Maharashtra, India.

SUPPORTING INFORMATION

MAI synthesis

10 ml of Hydro-iodic acid (HI) (57wt% in water, sigma Aldrich) was added drop-wise in 24ml of methyl amine (33 wt% in absolute ethanol, sigma Aldrich) at 0°C. The solution was then kept for stirring for 2 hours. Raw MAI powder product was then collected by drying the solution in a rotary evapourator at 50°C. Purification was performed by dissolving the raw product in 80ml ethanol and precipitating by the addition of 300ml diethyl ether. The purification process was repeated twice. The final product CH_3NH_3I was collected after drying in a vacuum oven at 60°C for 24 hours.

Fig. S1: UV-Visible absorption spectra of $CH_3NH_3PbI_3$ film exposed to solvent vapours for 15 minutes

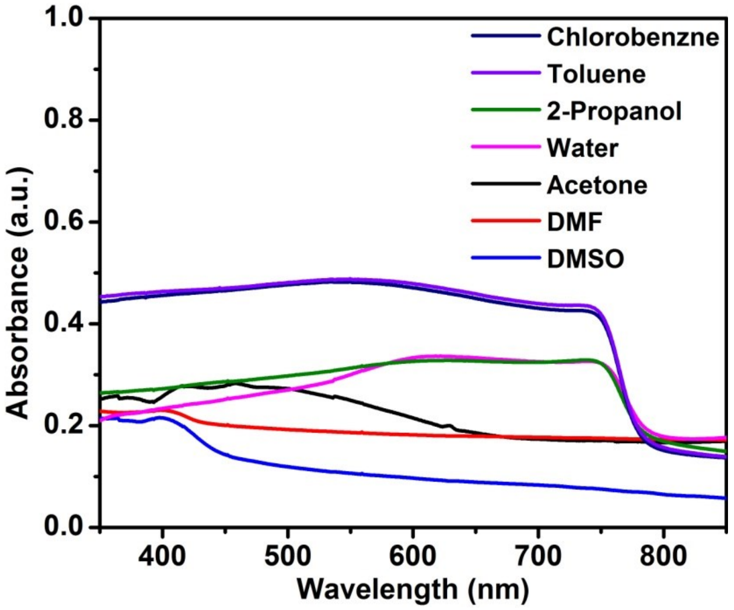

When $CH_3NH_3PbI_3$ films are exposed for 15 minutes the effect of polar aprotic solvent vapours is quite similar to those of when exposed for 30 minutes shown in manuscript. The effect of polar protic solvent vapours shown here is less than that of when exposed for 30 minutes. Nearly no changes were observed after exposing to nonpolar solvents for 15 minutes.

Fig. S2: Fourier Transformed Infra-Red spectra of as deposited $CH_3NH_3PbI_3$ and after exposing to vapours of a) Chlorobenzene, b) Toluene, c) 2-propanol, d) water, e) Acetone, f) DMF (includes spectrum of DMF and MAI+$PbCl_2$ in DMF), and g) DMSO solvents for 15 and 30 minutes.

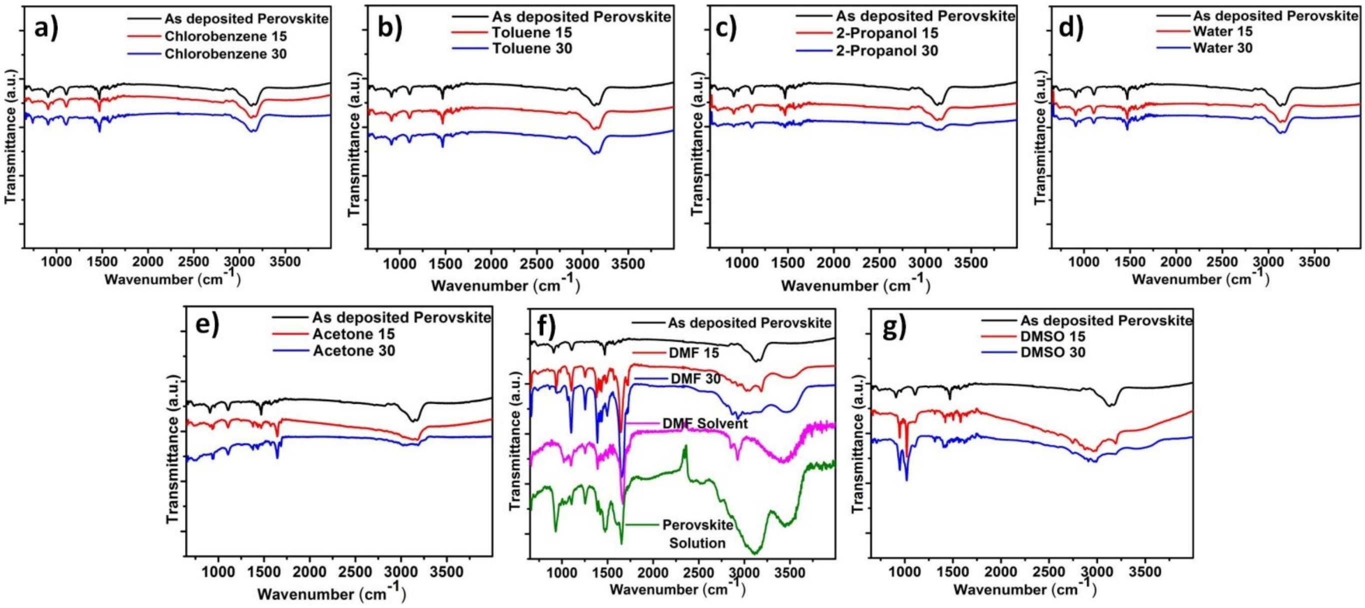

Fig. S3: Raman spectra of as deposited $CH_3NH_3PbI_3$ and after exposing to vapours of a) Chlorobenzene, b) Toluene, c) 2-propanol, d) water, e) Acetone, f) DMF and g) DMSO solvents for 30 minutes.

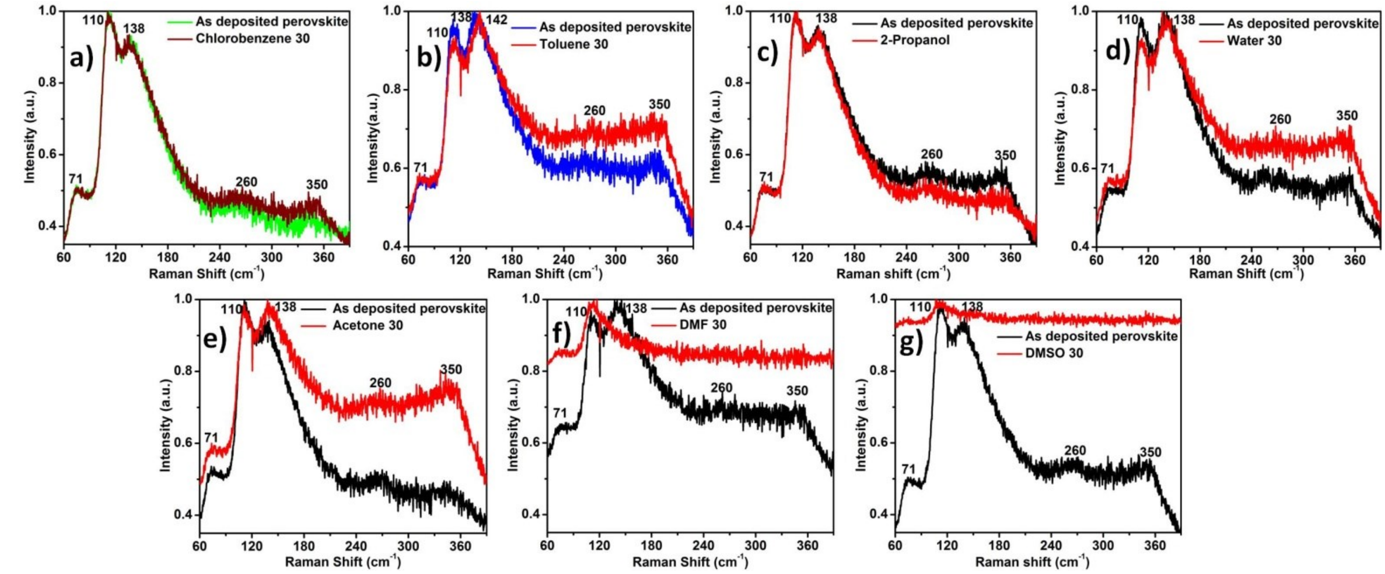

Supplement: RA-010-D0RA07926J-s001 [file RA-010-D0RA07926J-s001.pdf]
